# Detailed Process Analysis of Biobased Polybutylene Succinate Microfibers Produced by Laboratory-Scale Melt Electrospinning

**DOI:** 10.3390/polym13071024

**Published:** 2021-03-26

**Authors:** Maike-Elisa Ostheller, Naveen Kumar Balakrishnan, Robert Groten, Gunnar Seide

**Affiliations:** 1 Aachen-Maastricht Institute for Biobased Materials (AMIBM), Maastricht University, Brightlands Chemelot Campus, Urmonderbaan 22, 6167 RD Geleen, The Netherlands; m.ostheller@maastrichtuniversity.nl (M.-E.O.); naveen.balakrishnan@maastrichtuniversity.nl (N.K.B.); 2 Department of Textile and Clothing Technology, Niederrhein University of Applied Sciences, Campus Moenchengladbach, Webschulstrasse 31, 41065 Moenchengladbach, Germany; Robert.Groten@hs-niederrhein.de

**Keywords:** polybutylene succinate, fiber spinning, nonwoven, environmental sustainability, melt spinning, fiber production, electrospinning, melt electrospinning, process development, biomedical applications

## Abstract

Melt electrospinning is widely used to manufacture fibers with diameters in the low micrometer range. Such fibers are suitable for many biomedical applications, including sutures, stents and tissue engineering. We investigated the preparation of polybutylene succinate microfibers using a single-nozzle laboratory-scale device, while varying the electric field strength, process throughput, nozzle-to-collector distance and the temperature of the polymer melt. The formation of a Taylor cone followed by continuous fiber deposition was observed for all process parameters, but whipping behavior was enhanced when the electric field strength was increased from 50 to 60 kV. The narrowest fibers (30.05 µm) were produced using the following parameters: electric field strength 60 kV, melt temperature 235 °C, throughput 0.1 mL/min and nozzle-to-collector distance 10 cm. Statistical analysis confirmed that the electric field strength was the most important parameter controlling the average fiber diameter. We therefore report the first production of melt-electrospun polybutylene succinate fibers in the low micrometer range using a laboratory-scale device. This offers an economical and environmentally sustainable alternative to conventional solution electrospinning for the preparation of safe fibers in the micrometer range suitable for biomedical applications.

## 1. Introduction

Biocompatible and biodegradable polyesters are increasingly important in biomedical procedures, especially when used as sutures, bone fixation devices, plates, stents and screws, as well as tissue repair and tissue engineering matrices [1]. Polylactic acid (PLA) and polybutylene succinate (PBS) are widely available thermoplastic biopolymers that could replace petrochemical polymers in the future [2]. Biobased polyesters for medical applications are typically used in the form of films [3] or scaffolds in tissue engineering [4]. They can be manufactured by salt leaching [5], extrusion [6] or electrospinning [7]. 

Electrospinning is an efficient method for the manufacture of fibers ranging in diameter from a few micrometers to hundreds of nanometers [8,9,10]. Such fibers are beneficial because they combine an enormous surface area with high flexibility [11]. They are particularly useful for tissue engineering because fibers can be spun directly onto the surface of another material [12]. Further applications beyond the sphere of biomedicine include filtration and separation [13], as well as electronics and energy [14,15]. 

The global microfibers/nanofibers market was valued at US$477.7 million in 2016 and electrospinning was one of the most widely-used techniques for the production of such fibers [16]. The two major electrospinning methods are solution electrospinning and melt electrospinning, both of which involve the exposure of a liquid to a strong electric field as it flows through a capillary in order to draw out fibers [12]. The large potential difference (tens of kilovolts) applied to the liquid leads to the formation of a jet that undergoes whipping movements, which in turn cause stretching and ultimately the deposition of microscale or nanoscale fibers onto a collector [17]. Solution electrospinning uses polymer substrates dissolved in an organic solvent, which evaporates as the fiber is spun, whereas melt electrospinning uses a high-temperature molten polymer. The lower viscosity and higher electrical conductivity of polymer solutions enables solution electrospinning to produce thinner fibers than melt electrospinning. However, PLA and PBS must be dissolved in toxic solvents such as chloroform and dichloromethane, which can be carried over to the final product. To make the process more environmentally friendly, researchers are currently focusing on the use of more benign solvents such as formic acid and acetic acid [18,19]. Nevertheless, an additional solvent recovery step is required, increasing the overall process costs [12]. For example, the production of 1 kg of PLA fibers, when processed as a 10% solution, requires 10 L of solvent with only 90% recovered in a typical process [20]. In contrast, melt electrospinning does not require solvents, but the melt must be held at high process temperatures in order to facilitate extrusion [21]. Furthermore, the high viscosity and low conductivity of the melt yields fibers with a thicker average diameter than solution electrospinning [11]. Nevertheless, because melt electrospinning has no requirement for solvents, and therefore no need for a solvent recovery step, it is more cost effective and environmentally sustainable than solution electrospinning [22,23,24,25,26]. The wider adoption of melt electrospinning could help to reduce the environmental footprint of current industrial electrospinning processes for medical-grade fibers, but solution electrospinning remains the favored industrial process because it has already been scaled up (and is therefore more economical) and also produces finer fiber products [9].

Solution electrospinning with PBS and PLA has successfully yielded fibers with average diameters in the sub-micrometer range for biomedical applications such as wound dressings. Melt electrospinning has also been carried out with PLA, but the brittleness and low conductivity of the material hinder the production of nanofibers and their applications, and various machine and material modifications have been tested to overcome this [27,28,29,30,31,32,33,34,35,36,37]. Compared to PLA, PBS is more ductile with a lower glass transition temperature (below room temperature). Accordingly, modifications to improve the ductility of this polymer may not be necessary. 

A porous nonwoven PBS mesh with a low pore size has been produced by solution electrospinning and developed for filtration applications [31]. The evaluation of solution- electrospun microscale and nanoscale PBS fibers for biomedical applications confirmed that their high porosity and hierarchical structure offers sufficient mechanical properties for applications in wound healing and soft tissue engineering [4]. The PBS backbone has more polar functional units than PLA, which means that PBS may be more electrically conductive than PLA in its molten form. But, despite the advantages set out above, melt electrospinning with PBS has, to the best of our knowledge, not been attempted before. 

Here we report the preparation of the first melt-electrospun PBS microfibers using our single-nozzle laboratory-scale device. We investigated the influence of four different process parameters on fiber diameter: temperature, electric field strength, throughput and nozzle-to-collector distance. We measured the effect of temperature on the viscosity and electrical conductivity of PBS melts. We also examined the thermal properties of the polymer, its susceptibility to degradation during processing and the influence of process parameters on fiber crystallinity. Finally, statistical analysis was used to predict the parameter with the greatest influence on the fiber diameter.

## 2. Materials and Methods

### 2.1. Materials

Melt electrospinning was carried out using PBS fiber-grade resin (FZ78TM) produced by the polymerization of biobased succinic acid and 1,4-butanediol. The manufacturer (MCPP, Düsseldorf, Germany) reported the following specifications: a melt flow rate of 22 g/10 min at 190 °C and using a weight of 2.16 kg, and a crystalline melting temperature of 115 °C. The polymer was vacuum dried at 60 °C for 12 h before processing.

### 2.2. Methods

#### 2.2.1. Melt-Electrospinning Equipment

**Figure 1:**
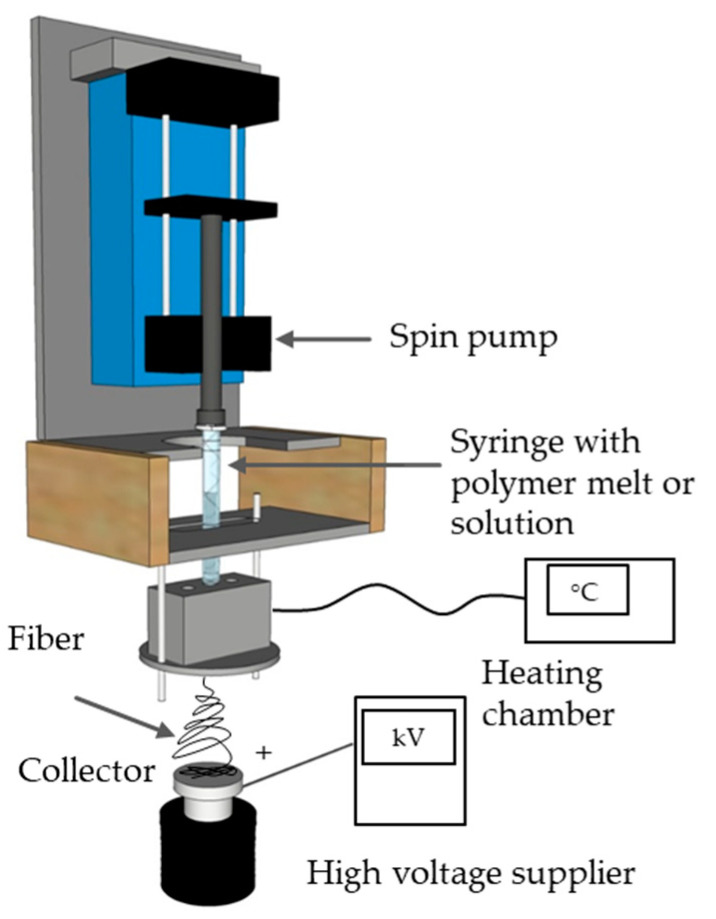
Schematic illustration of the laboratory-scale melt-electrospinning device.

For the evaluation of general processability and fiber formation characteristics, we used our laboratory-scale single-fiber melt-electrospinning device, including a temperature controller, high-voltage power supply, heating elements, syringe, pump and collector. The device was equipped with JCS-33A temperature process controllers (Shinko Technos, Osaka, Japan) and PT 100 platinum thermocouples (Omega Engineering, Deckenpfron, Germany) to control the melting temperature (Figure 1).

In previous studies with PBS, the reported melt-processing temperature was 190 °C [38]. Our laboratory-scale melt-electrospinning device does not use an extruder but uses a glass syringe instead. Because we are unable to apply any shear to make the polymer melt flow, we started with higher temperatures for the trials to compensate. Material flowed from the syringe at 205 °C but the viscosity was too high. Only temperatures > 235 °C supported constant and continuous fiber formation with a Taylor cone. Therefore, we carried out the trials with polymer melts at 235 and 265 °C. We used a KNH65 high-voltage generator (Eltex-Elektrostatik, Weil am Rhein, Germany) with a range of 6–60 kV. Potential differences of 50 and 60 kV were applied during the melt-electrospinning experiments, with positive voltage on the collector and a grounded spinneret. The collector was a 6 cm flat aluminum plate overlaid with a thin paperboard. Different distances between the spinneret and collector were tested, such as nozzle-to-collector distances of 8 and 10 cm. An 11 Plus spin pump (Harvard Apparatus, Cambridge, MA, USA) was used with delivery rates of 0.1 or 4 mL/min. A 2-mL glass syringe (Poulten & Graf, Wertheim, Germany) equipped with an additional metal orifice of 0.90 mm served as the spinneret nozzle. The experimental parameters are summarized in Table 1.

#### 2.2.2. Polymer Characterization

Thermogravimetric analysis (TGA) was carried out using a Q5000 device (TA Instruments, Asse, Belgium). We heated 5-mg PBS granules at a rate of 10 °C/min under nitrogen flowing at 50 mL/min until the temperature reached 700 °C. The temperatures at which 5% and 50% weight loss occurred were determined using TA universal analysis software. 

The rheological properties of PBS were characterized using a Discovery HR1 hybrid rheometer (TA Instruments) focusing on the frequency-dependent complex viscosity G*. We carried out a frequency sweep from 1 to 628 rad/s. For all experiments, we used a 25-mm plate-to-plate geometry with the distance set to 1000 μm, and the strain amplitude was maintained at 1%. Measurements were taken at temperatures of 145, 175, 205, 235 and 265 °C. The complex viscosity of PBS is presented at an angular frequency of 10 rad/s at different temperatures to facilitate comparative analysis. We made the rheological measurements 3 times at each temperature and we have presented the mean values along with the standard deviation for comparison.

**Figure 2:**
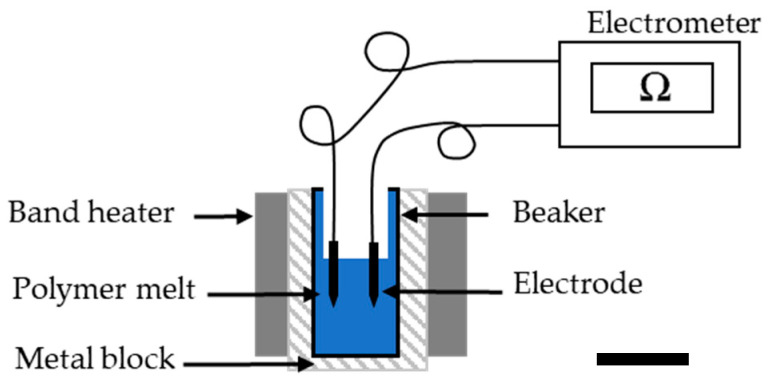
Electrometer apparatus for the analysis of electrical resistance in the PBS melt.

The electrical resistance of molten PBS was measured at the same temperatures as the rheological properties using a Keithley 617 electrometer (Tektronix, Beaverton, OR, USA). The polymer granules were melted using band heaters, and two electrodes (6 mm apart) were dipped in the melt and connected to the electrometer. The current flowing between the electrodes was measured at 10 V (Figure 2). The electrical resistance was measured 3 times at each temperature and the mean is presented for comparison.

#### 2.2.3. Characterization of PBS Fibers

Fiber diameters were measured using an Olympus BX53 microscope fitted with an Olympus DP26 camera (Olympus, Leiderdorp, The Netherlands). For each sample, the fiber diameter was measured 100 times in different positions based on the 50× magnified image.

Differential scanning calorimetry (DSC) was carried out using the Q2000 device focusing on changes to the glass transition temperature (Tg), melting temperature (Tm) and crystallinity (Xc) caused by different process parameters. DSC was applied to PBS granules as well as fibers with the smallest and largest diameters. Any change in fiber crystallinity during spinning is typically caused by the drawing process. Therefore, we selected samples that had undergone the most drawing (smallest diameter) and the least drawing (biggest diameter) to compare with the PBS granules. No change in crystallinity indicated that the process parameter did not influence the physical properties of PBS. We used a temperature range of −40 to +300 °C and a heating rate of 10 °C/min. The melt enthalpy of 100% crystalline PBS was considered to be 110.3 J/g [39]. For each sample, we made 3 measurements and the mean values are presented.

The effect of processing on the molecular weight of PBS was determined by gel permeation chromatography (GPC) using a 1260 Infinity System (Agilent Technologies, Santa Clara, CA, USA). We used hexafluor-2-isopropanol (HFIP) containing 0.19% sodium trifluoroacetate as the mobile phase, flowing at a rate of 0.33 mL/min. GPC was used to compare PBS granules with fibers spun at the lowest throughput at different processing temperatures (235 and 265 °C). By testing the low-throughput samples (longest dwell time), we were able to conclude that no degradation under these conditions would infer the absence of degradation at higher throughputs. Solutions were prepared by dissolving 5-mg samples in HFIP for ~2 h before passing through a 0.2-µm polytetrafluoroethylene filter and injecting them into a modified silica column filled with 7-µm particles (Polymer Standards Service, Mainz, Germany). The relative molecular weight (Mw), number average molar mass (Mn), and polydispersity index (PDI) were determined using refractive index detectors calibrated with a standard polymethyl methacrylate polymer (1.0 × 105 g/mol). We performed GPC analysis 3 times with each sample and presented the mean values for comparison.

#### 2.2.4. Statistical Analysis

We used a full factorial design with four factors and two levels (Table 1) for multi-way analysis of variance (ANOVA) in SPSS (IBM, New York, NY, USA) to determine the statistical significance of each parameter with a general univariate analysis. We tested the effect of independent factors (temperature, electric field strength, throughput and the distance between nozzle and collector) and their interactions on the fiber diameter. We also compared effect strength η^2^ values of different process parameters to determine which exerted the greatest influence.

## 3. Results and Discussion

### 3.1. Thermal Analysis of the Polymer

**Figure 3:**
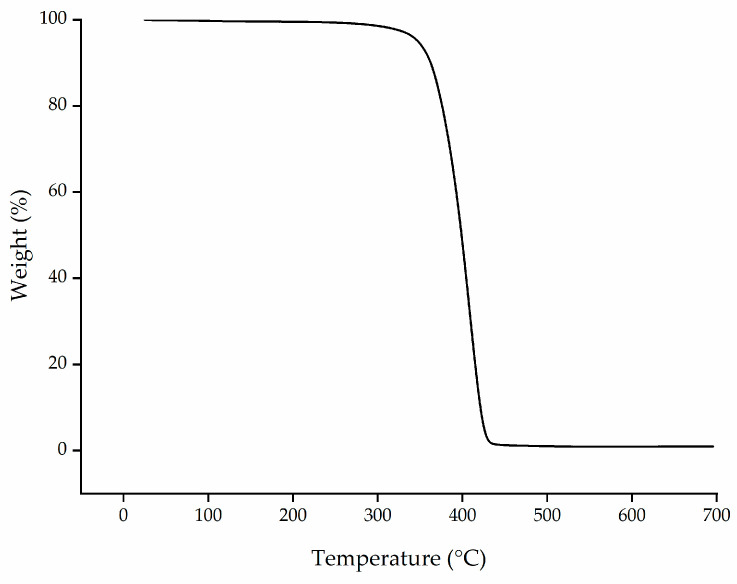
Thermogravimetric analysis (TGA) thermogram showing the temperature-dependent degradation profile of PBS granules.

TGA revealed that PBS granules undergo single-step degradation (Figure 3), with 5% weight loss at 347 °C and 50% weight loss at 398 °C, in agreement with previous reports [40]. The processing temperatures we selected are lower than the degradation temperatures determined by TGA.

### 3.2. Effect of Temperature on Viscosity

**Figure 4:**
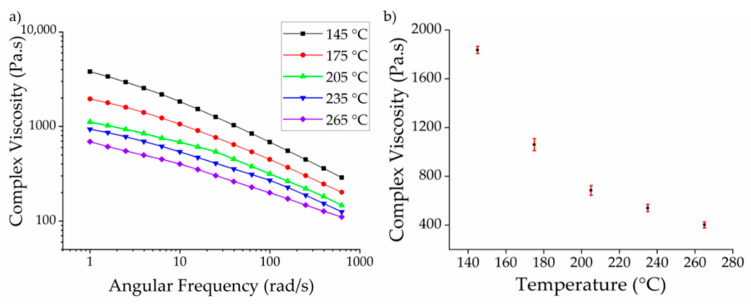
(**a**) Rheogram showing the complex viscosity of PBS melts with increasing angular frequency at different temperatures; (**b**) Complex viscosity of PBS as a function of temperature at an angular frequency of 10 rad/s. Data are means with standard deviation (*n* = 3).

The complex viscosity of PBS was plotted as a function of angular frequency at different temperatures (Figure 4a). This revealed that the melts approach a plateau of Newtonian behavior at low angular frequencies, but non-Newtonian (pseudoplastic) behavior is observed as the angular frequency increases. Accordingly, the complex viscosity declines sharply, as previously reported for PBS [41]. Entanglements and chain interactions such as van der Waals forces and hydrogen bonds in the polymer can hinder the polymer flow. At lower shear, the polymer chains move so slowly that these interactions do not impede the flow and the shear is not sufficient to break these interactions. Therefore, we observed Newtonian behavior, where viscosity is independent of shear. Such interactions can be broken by increasing the shear and/or the temperature. As the shear rate increases and the interactions are broken, the polymer chains become oriented in the direction of shear making the polymer flow easier. The declining complex viscosity we observed with increasing angular frequency thus reflected the corresponding increase in shear. To visualize the effect of temperature on the complex viscosity, the complex viscosity of PBS as a function of temperature is shown in Figure 4b at an angular frequency of 10 rad/s.

As anticipated, the complex viscosity of PBS declined with increasing temperature. There are two potential explanations for this observation. First, as set out above, the entanglements and chain interactions are broken at high temperatures, facilitating the flow of the polymer chains. Similar results have been reported for PLA [42]. We found that the complex viscosity of PBS declined by ~42% when the temperature increased from 145 to 175 °C, and by ~35% when the temperature increased from 175 to 205 °C. However, as we move further from the melting point (110 °C), there is a less significant decline in viscosity with increasing temperatures. The decline in viscosity was only ~20% when the temperature increased from 205 to 235 °C and a similar value was observed when the temperature increased from 235 to 265 °C, perhaps because most of the chain interactions and entanglements in the polymer are already broken at 205 °C so increasing the temperature has no further effect. The second potential explanation is the degradation of the polymer at higher temperatures, which we investigated by GPC (Section 3.4).

### 3.3. Effect of Temperature on Electrical Conductivity

**Figure 5:**
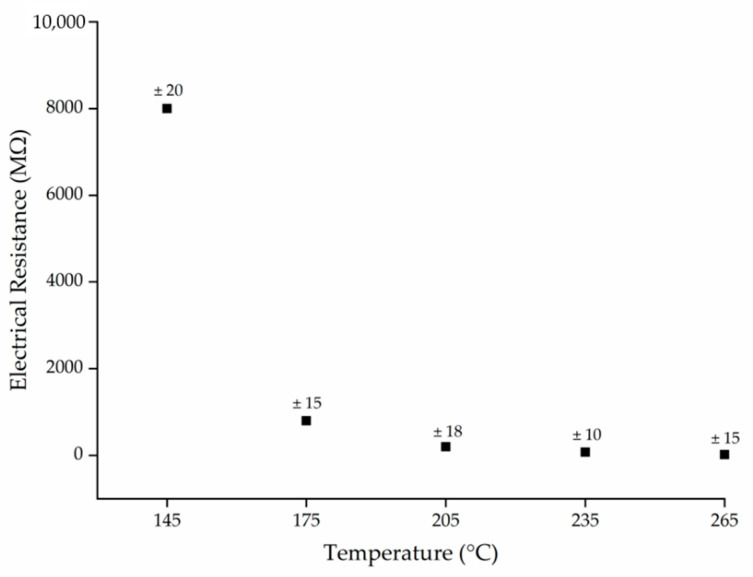
The electrical resistance (MΩ) of PBS melts as a function of temperature. Data are means with ± standard deviation (*n* = 3).

The electrical resistance of PBS was measured at melt temperatures of 145, 175, 205, 235 and 265 °C to determine the effect of temperature on conductivity. We found that higher temperatures generally reduced the electrical resistance (Figure 5). The resistance of the polymer melt was ~8 GΩ at 145 °C, but increasing the temperature by 30 °C to 175 °C reduced the electrical resistance by 10-fold. Further reductions, albeit much smaller in magnitude, were observed at 205, 235 and 265 °C. The lowest electrical resistance of 20 MΩ was observed at 265 °C. 

Polymers are insulators and the electrical conductivity is intrinsically dependent on the temperature [43]. For polymers with polar functional groups (such as PBS), higher temperatures increase the mobility of the polymer chains, leading to ionic conduction via the polar groups and micro-Brownian motion [44]. The observed trend in electrical resistance as a function of temperature supports the rheological data (Figure 4b). The change in viscosity of the polymer reached a plateau at temperatures exceeding 205 °C. Increasing the temperature beyond this point did not substantially increase the mobility of the polymer chains. The change in electrical resistance followed the same trend, further supporting the hypothesis that ionic conduction in PBS is mediated by the mobility of the polymer chains.

### 3.4. Molecular Weight of the Melt Electrospun Fibers

**Figure 6:**
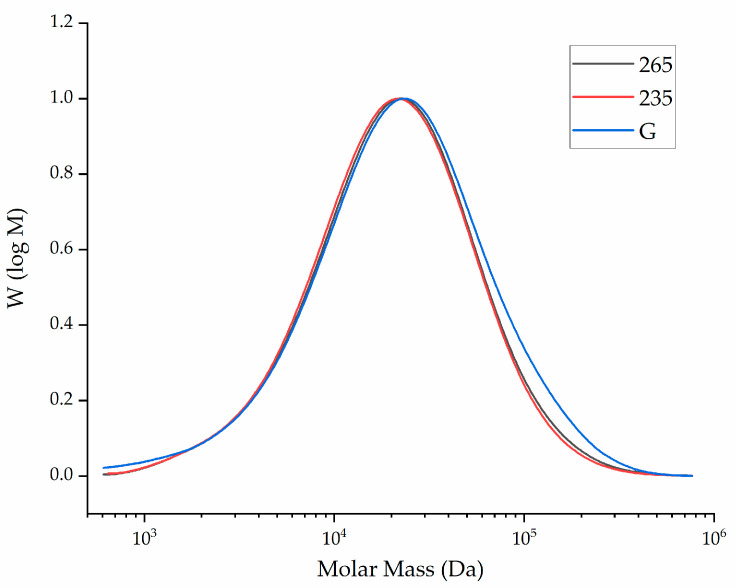
The molar mass distribution curve of unprocessed PBS granules (G) and PBS fibers processed at 235 and 265 °C.

The GPC curves of unprocessed PBS granules and PBS fibers processed at 235 and 265 °C are presented in Figure 6.

**Figure 7:**
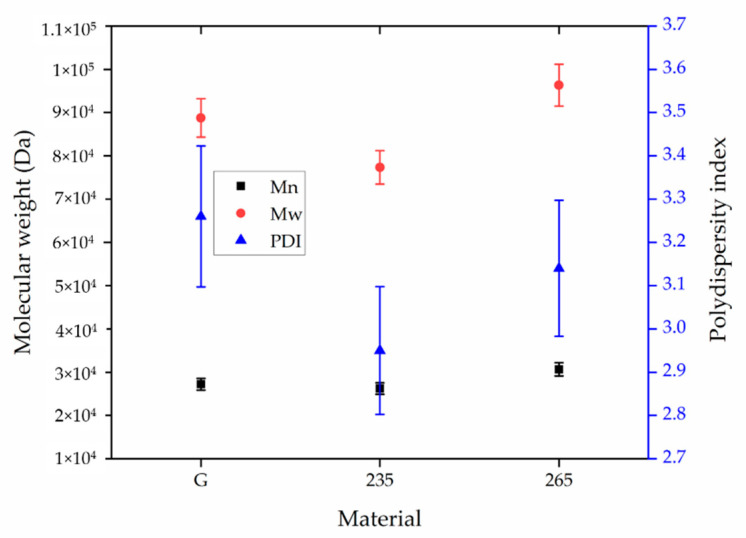
Weight average relative molecular weight (Mw), number average molar mass (Mn) and polydispersity index (PDI) of unprocessed PBS granules (G) and PBS fibers processed at 235 and 265 °C. Data are means with standard deviation (*n* = 3).

The relative Mw, Mn and PDI of the same samples are presented in Figure 7.

The Mn, Mw and PDI of unprocessed PBS granules were 27,210 Da, 88,760 Da and 3.26, respectively. The Mn, Mw and PDI of the PBS fibers processed at 235 °C were 26,210 Da, 77,350 Da and 2.95, respectively. The corresponding values for the fibers processed at 265 °C were 30,660 Da, 96,340 Da and 3.14, respectively. The results indicated that processing PBS at these specific temperatures resulted in no significant change in the MW, Mn or PDI. This confirms that any reduction in viscosity is explained by the higher temperature increasing chain mobility and not by polymer degradation.

### 3.5. Fiber Diameter and Distribution

We next investigated the processability of PBS and the effect of temperature, electric field, throughput and nozzle-to-collector distance on the fiber diameter. When the polymer melt droplet becomes charged in a field of sufficient strength, the electrostatic repulsion is strong enough to overcome the surface tension and stretch the droplet. When this charge becomes higher than a certain threshold, a jet known as a Taylor cone erupts from the polymer droplet [45]. We observed the formation of a Taylor cone under all the process conditions we tested. 

**Figure 8:**
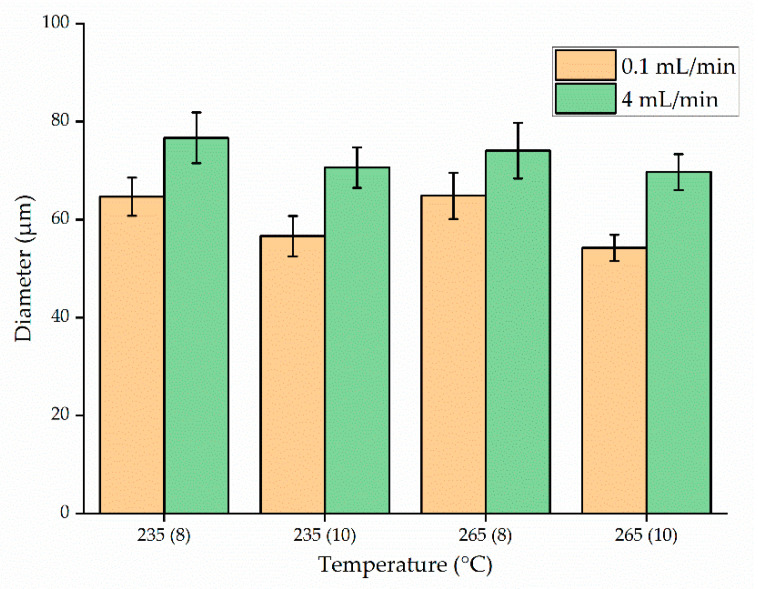
Diameter of PBS fibers at polymer melt temperatures of 235 or 265 °C, throughputs of 0.1 or 4 mL/min, a nozzle-to-collector distance of 8 or 10 cm, and the electric field strength set to 50 kV. The *x*-axis shows the temperature with the nozzle-to-collector distance in parentheses. Data are means with standard deviations (*n* = 100).

Figure 8 shows the average fiber diameter (±standard deviation) produced at spinneret temperatures of 235 and 265 °C, with nozzle- to-collector distances of 8 and 10 cm, throughputs of 0.1 and 4 mL/min and the electric field strength set to 50 kV. Reducing the throughput from 4 to 0.1 mL/min generated finer fibers at both temperatures. Because the polymer melt emerging from the nozzle is pulled down by gravity and by its interaction with the electric field, we hypothesized that reducing the throughput gives more time for the polymer to interact with the electric field, increasing the probability of whipping behavior. The fiber diameter produced at a throughput of 0.1 mL/min was also significantly reduced by increasing the nozzle-to-collector distance from 8 to 10 cm. For example, at 265 °C and a throughput of 0.1 mL/min, increasing the distance from 8 to 10 cm reduced the average fiber diameter by 15.6% (from 65 to 54 µm). As proposed above, increasing the nozzle-to-collector distance would also give the fiber more time to interact with the electric field, thereby promoting whipping behavior. But if the collector were placed too far from the nozzle, there would be less interaction with the electric field leading to thicker fibers. Under similar conditions with a throughput of 4 mL/min, the same change in distance achieved only a 6.7% reduction in fiber diameter. A similar trend was observed at 235 °C. When increasing the throughout from 0.1 to 4 mL/min, the material flow increased 40-fold. Given this higher material flow, it is likely that increasing the nozzle-to-collector distance by 2 cm is not sufficient to provide enough additional time for the fibers to interact with the field.

**Figure 9:**
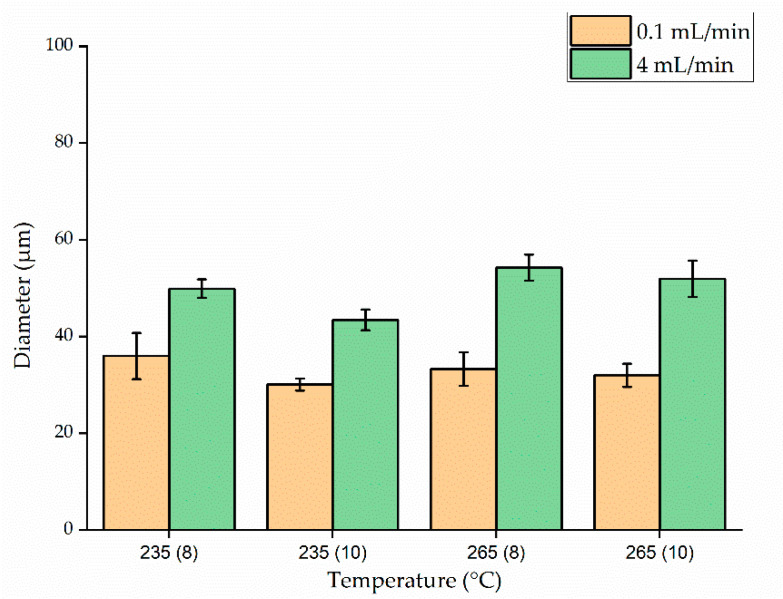
Diameter of PBS fibers at polymer melt temperatures of 235 or 265 °C, throughputs of 0.1 or 4 mL/min, a nozzle-to-collector distance of 8 or 10 cm, and the electric field strength set to 60 kV. The *x*-axis shows the temperature with the nozzle-to-collector distance in parentheses. Data are means with standard deviations (*n* = 100).

We found that the temperature had no significant effect on fiber diameter compared to throughput or nozzle-to-collector distance. For example, the mean fiber diameter at a throughput of 0.1 mL/min and a nozzle-to-collector distance of 10 cm was 56 µm at 235 °C and 54.22 µm at 265 °C, the latter being the narrowest fiber produced at 50 kV. However, much finer fibers were produced when we increased the electric field strength to 60 kV (Figure 9). Although Taylor-cone formation and whipping behavior occurred under all the process parameters we tested, the whipping behavior was enhanced at 60 kV. Accordingly, the average fiber diameter at a temperature of 235 °C, a throughput of 0.1 mL/min, and a nozzle-to-collector distance of 10 cm was reduced by 46% (from 56 to 30 µm) in the stronger electric field. This result is consistent with previous studies [2].

The trend toward narrower fibers at a lower process throughput was maintained at the higher voltage. By reducing the throughput from 4 to 0.1 mL/min, the average fiber diameter decreased by 30% (from 50 to 35 µm) at a temperature of 235 °C and a nozzle-to collector distance of 8 cm. This phenomenon was observed regardless of the processing temperature or nozzle-to-collector distance.

**Figure 10:**
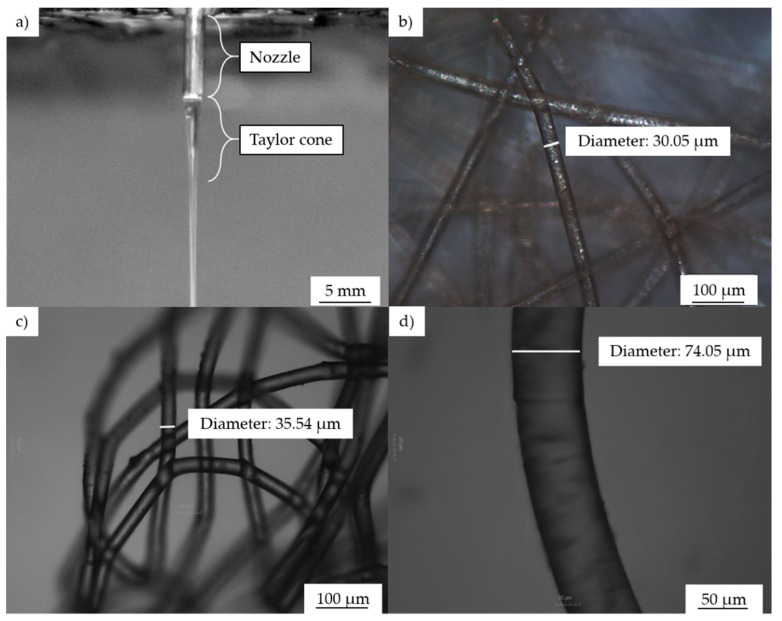
Taylor cone formation during melt electrospinning and images of the resulting fibers. (**a**) Taylor cone formation. (**b**–**d**) Microscopy images of PBS electrospun fibers under different processing conditions. (**b**) Temperature: 235 °C, throughput: 0.1 mL/min, electric field: 60 kV, nozzle-to-collector distance: 10 cm. (**c**) Temperature: 265 °C, throughput: 0.1 mL/min, electric field: 60 kV, nozzle-to-collector distance 8 cm. (**d**) Temperature: 265 °C, throughput: 4 mL/min, electric field: 50 kV, nozzle-to-collector distance: 8 cm.

When we compared fiber diameters as a function of temperature while keeping the remaining parameters constant, we found that temperature had no significant influence. An interesting phenomenon observed during high-throughput processing at 265 °C and 60 kV was that changing the nozzle-to-collector distance did not affect the fiber diameter. The average fiber diameter at a throughput of 4 mL/min was 33 and 32 µm when the nozzle-to-collector distance was 8 and 10 cm, respectively. The viscosity and resistance of PBS were also marginally lower at 265 than at 235 °C. As the material comes through the nozzle, fibers are formed due to a combination of gravitational pull and the electric field. One hypothesis to explain the effect observed at the high temperature in the strongest electric field is that the material flows so quickly under these conditions that increasing the nozzle to collector distance by 2 cm does not increase the whipping behavior and thus has no significant effect on the fiber diameter. When the electric field strength was 60 kV, the finest fibers (30.05 µm) were produced at 235 °C with a throughput of 0.1 mL/min and a nozzle-to-collector distance of 10 cm. The Taylor cone formation observed during melt electrospinning and microscopy images of the resulting PBS fibers produced are presented in Figure 10. 

The melt electrospinning of PLA under similar conditions (identical apparatus, electric field strength 60 kV, nozzle-to-collector distance 10 cm, throughput 4 mL/min, temperature 300 °C) generated fibers with an average diameter of 112.5 µm, whereas at a lower temperature of 235 °C, the diameter of PBS fiber produced were 43.42 µm (61% lower). These results suggest that PBS is more suitable as a material for melt electrospinning than PLA [34].

**Figure 11:**
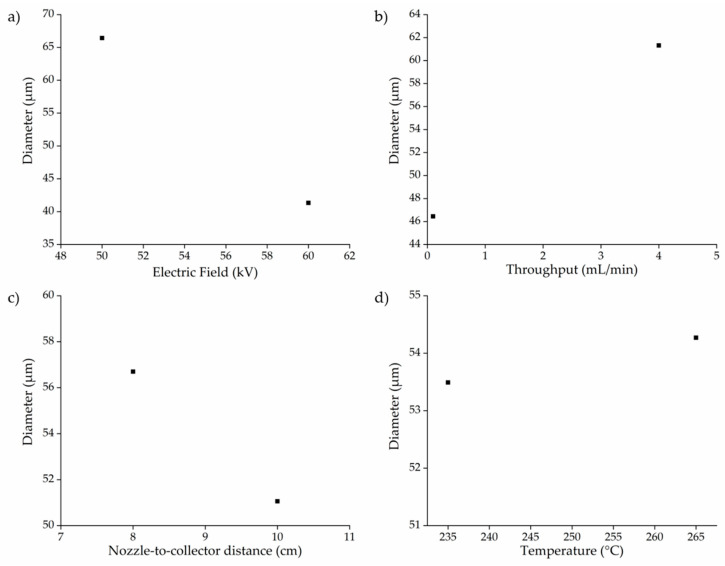
Variations in PBS fiber diameter as a function of different process parameters. (**a**) Electric field strength vs. diameter, (**b**) throughput vs. diameter, (**c**) nozzle-to-collector distance vs. diameter, (**d**) temperature vs. diameter.

Statistical analysis of the datasets in Figure 8 and Figure 9 by multi-way ANOVA revealed the statistical significance of differences in fiber diameter as a function of the process parameters (temperature, throughput, electric field strength, and nozzle-to-collector distance) and their interactions. The relationships between individual process parameters (factors) and the average fiber diameter are summarized in Figure 11. Narrower fibers were produced by reducing the process throughput or by increasing the electric field strength or nozzle-to-collector distance. Higher temperatures also tended to produce narrower fibers but the effect of this parameter was not significant.

**Figure 12:**
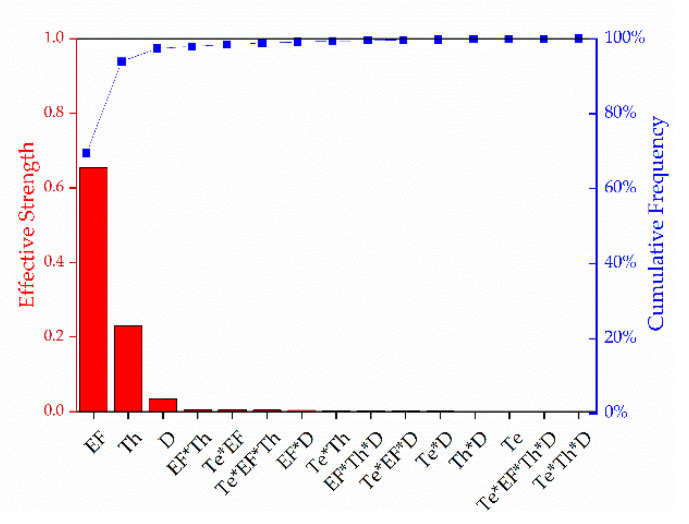
Pareto chart representing the effective strength of the factors we tested and their interactions. EF = electric field, Th = throughput, D = nozzle-to-collector distance, Te = temperature.

The effective strength of the various factors we considered and their interactions are summarized below in Figure 12.

The significance level of the factors and their interactions are presented in Table 2.

Statistical analysis revealed that the electric field, throughput, and nozzle-to-collector distance all had a significant impact (*p* < 0.05) on the diameter of the fiber whereas temperature was not statistically significant (*p* > 0.05). This agrees with the data reported earlier. Under fixed conditions of a 0.1 mL/min throughput, a 10 cm nozzle-to-collector distance and a 50 kV electric field, the diameter of the fiber obtained only changed from 56 µm at 235 °C to 54.22 µm at 265 °C. The possible hypothesis for this was explained using the changes observed in the rheological behavior and electrical conductivity of the polymer as a function of temperature. 

We also observed statistically significant interactions (*p* < 0.05) between the factors temperature and electric field, temperature and throughput, electric field and throughput, electric field and nozzle-to-collector distance, and finally temperature, electric field and throughput. As shown in Figure 12, the electric field has the strongest influence on the fiber diameter, followed by throughput, then nozzle-to-collector distance. This also agrees with the data presented earlier. Among all the factors we tested, increasing the electric field from 50 kV to 60 kV led to the most substantial reduction in fiber diameter.

### 3.6. Thermal Properties of the Electrospun Fibers

**Figure 13:**
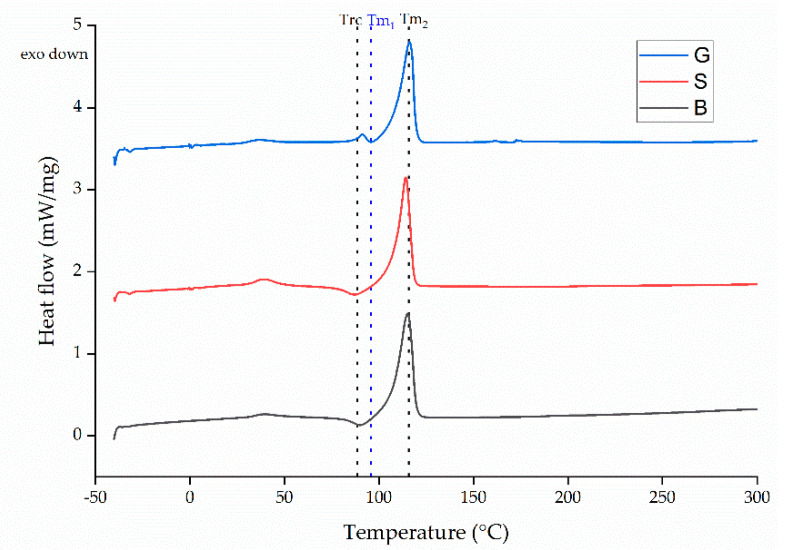
DSC thermogram of PBS granules (G) and PBS fibers with the smallest (S) and biggest (B) diameters we achieved using our processing conditions.

The DSC thermograms of PBS granules and PBS fibers with the highest diameter (76.68 µm) and lowest diameter (30.05 µm) produced under our processing conditions are presented in Figure 13. 

The Tg, Tm and Xc values are summarized in Table 3.

The DSC thermograms of the granules and both fibers featured a melting peak (Tm_2_) at ~110 °C, which is also the PBS melting point reported by the manufacturer. The values we observed agree with those reported in previous studies [46]. Another small peak was observed at ~50 °C, possibly representing an additive such as a plasticizer used to improve the processability of PBS. The PBS granules feature a second melting peak just below 100 °C (Tm_1_). This is mirrored by exothermic peaks in the thermograms for each of the fibers (Trc) possibly associated with the recrystallization (or recrystallization and melting) of PBS. The crystallization process occurring during fiber formation can involve the formation of crystals differing in size and containing various defects. As the PBS is heated, these small crystals or in some cases, crystal defects, can melt and recrystallize or combine to form larger crystals, which melt again at the higher temperature. This could also be from amorphous polymer chains, due to higher mobility at these temperatures, forming a structure that is more ordered and therefore leading to crystallization. Similar observations have been reported in earlier studies [47]. The enthalpies of melting of the melting peaks at 35 °C were not considered for the calculation of Xc because they are thought to correspond to the melting of an additive. However, the enthalpy of recrystallization was taken into account and subtracted from the melting enthalpy to calculate the total crystallinity of PBS. In the case of PBS granules, the enthalpy of melting from the first melting peak (Tm_1_) was taken into consideration and added to the enthalpy obtained from the Tm_2_ to obtain the Xc value. The Xc of all samples remained constant at ~57%. Our DSC results therefore suggest that the range of process parameters we tested did not affect the physical properties of the PBS fibers.

## 4. Conclusions

We have investigated the melt electrospinning of PBS microfibers using a single-nozzle laboratory-scale device, testing different process parameters (temperature, electric field strength, throughput and nozzle-to-collector distance) to determine their effect on fiber diameter. To the best of our knowledge, we have reported the first melt-electrospun PBS fibers produced in the low micrometer range. We observed the formation of a Taylor cone followed by continuous fiber deposition throughout the range of process parameters we tested. The coarsest fibers (diameter = 74.05 µm) were produced at 265 °C with an electric field strength of 50 kV, a throughput of 4 mL/min and a nozzle-to-collector distance of 8 cm. The whipping behavior was enhanced by increasing the electric field strength from 50 kV to 60 kV. The finest fibers (diameter = 30.05 µm) were produced at 235 °C and 60 kV, with a throughput of 0.1 mL/min and a nozzle-to-collector distance of 10 cm. 

We found that low-throughput melt electrospinning (0.1 mL/min) reduced the fiber diameter at both temperatures and field strengths we tested. The effect of changing the nozzle-to-collector distance from 8 to 10 cm was also more significant at low throughput. It is likely that increasing the nozzle-to-collector distance during a low-throughput process allows more time for whipping behavior, thus stretching the melt and generating narrower fibers. In contrast, the fast flow of the polymer during the high-throughput process means that a 2-cm increase in the nozzle-to-collector distance does not have a significant effect on whipping behavior and thus on the fiber diameter.

Multi-way ANOVA revealed that three factors (electric field strength, throughput, and nozzle-to-collector distance) had a significant effect on fiber diameter, whereas the effect of temperature was nonsignificant when the other factors remained constant. Our rheological and conductivity data showed that the change in viscosity and electrical conductivity was not significant within the temperature range we considered, and this may explain the results of the ANOVA test. Based on the effect strength η^2^, the electric field strength was the factor with the greatest influence on the fiber diameter. 

By varying four process parameters, we reduced the average diameter of our melt electrospun PBS fibers by 59% (from 74.05 to 30.05 µm). The narrowest fibers we produced were significantly finer than those we previously generated from PLA using the same experimental setup. Our results suggest that PBS could replace PLA and other polymers for the melt electrospinning of biomedical fibers. This provides a sustainable alternative to solution electrospinning, which is normally used to manufacture fibers for biomedical applications.

## Figures and Tables

**Table 1:** Experimental parameters used for the melt electrospinning of polybutylene succinate (PBS) fibers.

Temperature (°C)	Electric Field (kV)	Nozzle-to-Collector Distance (cm)	Throughput (mL/min)
235	50	8	0.1
	50	8	4
	50	10	0.1
	50	10	4
	60	8	0.1
	60	8	4
	60	10	0.1
	60	10	4
265	50	8	0.1
	50	8	4
	50	10	0.1
	50	10	4
	60	8	0.1
	60	8	4
	60	10	0.1
	60	10	4

**Table 2:** Significance level of the factors we tested and their interactions. EF = electric field, Th = throughput, D = nozzle-to-collector distance, Te = temperature.

Factor	Significance Level (*p*)
EF	<0.001
Th	<0.001
D	<0.001
Te	0.209
Te*EF	<0.001
Te*Th	0.015
Te*D	0.113
EF*Th	0.001
EF*D	0.009
Th*D	0.183
Te*EF*Th	0.003
Te*EF*D	0.055
Te*Th*D	0.439
EF*Th*D	0.051
Te*EF*Th*D	0.343

**Table 3:** The melting temperature (Tm), re-cooling temperature (Trc) and degree of crystallinity (Xc) of PBS granules (G) and PBS fibers with the smallest (S) and biggest (B) diameters we achieved using our processing conditions. Data are means with ± standard deviations (*n* = 3).

Material	Tm_1_ (°C)	Trc (°C)	Tm_2_ (°C)	Xc (%)
G	91.1 ± 2	-	116.3 ± 2	59.97 ± 1
S	-	88.6 ± 3	114.2 ± 1	55.6 ± 1
B	-	90.4 ± 2	115.2 ± 2	58.9 ± 1

## Data Availability

The datasets used and/or analyzed during this study are available from the corresponding author on reasonable request.
